# Health and education needs of primary school children with Down Syndrome in England: What can we learn from linked administrative data?

**DOI:** 10.23889/ijpds.v9i5.2695

**Published:** 2024-09-18

**Authors:** Julia Shumway, Ruth Gilbert, Bianca De Stavola, Ania Zylbersztejn

**Affiliations:** 1 UCL Great Ormond Street Institute of Child Health; 2 NIHR Great Ormond Street Hospital Biomedical Research Centre

## Objective

Children with Down syndrome (DS) have high rates of chronic health conditions (CHCs) which can affect special education needs and primary school placement (mainstream or special). We assessed timing and type of CHC by year-specific primary school placement (ages 5 to 11 years).

## Approach

We followed two cohorts: first, a birth cohort of all children with DS born from 2003 to 2020 in England using hospital administrative data; second, a sub-cohort with linked hospital and education records who enrolled in state-funded primary schools in England at age 5 from 2009/10 to 2013/14. We calculated the cumulative incidence of CHCs and multimorbidity (defined as distinct CHCs affecting two or more body systems) from birth through age 11, accounting for variable follow-up and the competing risk of death. We assessed multimorbidity by school placement over time.

## Results

The birth cohort included 11,478 children with DS (prevalence: 0.11%). By age 5, 6.2% of children had died, 80.8% had at least one recorded CHC, and 53.4% had multiple CHCs. Corresponding figures by age 11 were 6.9%, 87.4% and 65.9%, respectively.

85% of children with DS alive at school entry linked to a school record. 72% were enrolled in mainstream primary school at age 5, decreasing to 62% by age 7, and 54% by age 11.

## Conclusions

Most children with DS have multiple hospital-recorded CHCs. Nearly three-quarters start mainstream primary school, but just over half remain by age 11.

## Implications

Parents, clinicians, and educators will benefit from understanding relationships between multimorbidity and school placement.

